# Energy security for socio-economic and environmental sustainability in Pakistan

**DOI:** 10.1016/j.heliyon.2018.e00854

**Published:** 2018-10-15

**Authors:** Shahzada M. Naeem Nawaz, Shahzad Alvi

**Affiliations:** a Punjab Economic Research Institute, Lahore, Pakistan; b School of Social Sciences and Humanities, National University of Sciences and Technology, Islamabad, Pakistan

**Keywords:** Economics, Energy

## Abstract

One of the prime concern of policy makers should be to ensure energy security in the country. The case of Pakistan is interesting in a sense that with the growing age of the country, the increasing reliance on imported sources of energy is resulting in huge demand-supply gap which is evident in suppressed demand for electricity and natural gas. It has social, economic and environmental consequences. Most of the countries in the world have shifted their focus from imported to indigenous resources which are more often cheap, environmental friendly and provide competitiveness**.** The study reveals, through exploring secondary data, descriptive analysis, Johanson Cointegration and Granger Causality test results based on error correction model, energy insecurity is damaging for environment and socio-economic conditions. The study suggests policy makers to move toward long run energy security to get environmental and socio-economic sustainability in the country through minimizing reliance on imported energy and promoting energy efficiency.

## 1 Introduction

Energy security is a question of national strategy since World War I when Churchill shifted the power source of the British Navy to oil from coal. However, after the oil price shock of 1973, the concept of energy security has changed in a way to less reliance on uncertain oil supplies which has socio-economic and environmental consequences as well. Moreover, large reliance on imported energy would result in large foreign exchange expenditures, expensive energy, high cost of production, less competitiveness, high CO_2_ emissions, psychological cost and much more. Reducing dependence on imported fuels and diversifying energy sources for production of electricity is an important policy direction to enhance energy security which also leads to reduction in emissions and promote economic development. Implementation at small and medium scale is an important feature of renewables which can also be adopted as off-grid solution [1]. Having this perspective in mind, the world has changed its focus from imported expensive energy sources to cheaper renewable sources, bringing socio-economic and environmental sustainability in the country.

Energy security is also a matter of concern to power blackout, meaning thereby electricity supply must be uninterrupted whereas the key lies in diversification of energy resources along with optimal utilization of indigenous resources. Robustness of electricity supply is one of the important perspective to increase reliability which depends on the capacity or adequacy to meet the demand and security which measures responsiveness of facilities to shocks within the system [2, 3]. It is really a challenge for the developing economies, being at initial phase of industrialization, to ensure energy security primarily because of limited financial sources and prevalence of appropriate policies. Thus, reliability, affordability and accessibility along with sustainability are four major components of energy security for any country.

Economies around the globe are facing high energy demand to achieve sustained economic growth. However, the challenge is not only to meet the rising demand but also to put less reliance on the depleting fossil fuels which cause damaging environmental effects. Volatile price dynamics of fossil fuels and widening demand-supply gap of electricity calls for cost effective, environment friendly and reliable energy resources. These factors result in an increasing interest in developing renewable resources. Policy makers globally have largely recognized the significance of relationship between energy and economic progress. That being the case, it's an agreed fact that economic development and energy reinforce each other.

According to [4], policy makers usually take into consideration the social and economic aspects of energy security for affordability and accessibility of services. Moreover, sustainability of energy supply also requires reduction of emissions to control the absorption capacity of the environment. Extraction or utilization of non-renewable and renewable sources is also a policy issue to control the generation rates [5]. Affordable and sustainable energy supplies not only bring prosperity for the population at large but also help eradicate poverty through various direct and indirect channels. Pakistan's Vision 2025, while accepting energy security as a challenge, aims to achieve Sustainable Development Goal 7 “Ensure access to affordable, reliable, sustainable and modern energy for all” by 2025. Pakistan is facing worst ever shortfall of electricity supply since year 2007 whereas the reliance on imported thermal resources is an increasing phenomenon since 1990s. Slowdown of economic growth through various channels, such as, social cost and increasing trend in CO_2_ emissions are the consequences but the policymakers are still insisting on imported sources to fulfil the energy needs.

Increasing requirement of hydrocarbons cannot be avoided with the growing population but its pace can be slowed down through producing power from renewable sources which are sufficiently available domestically. Pakistan is among the top 10 countries facing severe energy crisis [6]. It is meeting 85% of its total petroleum products demand through imports. Out of total demand of 23.6 million metric ton, around 30% of imports, are meant to produce power only. Historically, electricity diversity is downward sloping and CO2 emissions has increasing trend. Pakistan must focus on the modern sources of energy because of their crucial importance in meeting Sustainable Development Goal (SDGs) and making the investment climate better. Accordingly, ensuring fair development process without undermining welfare for future generation should be the criterion. Whereas, more reliance on modern energy use may enable the poor population of developing countries to participate in productive use rather consumptive use leading to improved living conditions [7, 8].

Access to electricity, hence the energy security up to some extent at least, has already been included as a component of Multidimensional Poverty index by the United Nations Development Programme in 2010. Access to electricity and least reliance on imported energy resources should be a major component of national strategy. According to Ban Ki-Moon, “Energy is the golden thread that connects economic growth, social equity, and environmental sustainability.” Due to worst crisis being faced by Pakistan, social and economic development is in declining phase and conventional sources are not enough to meet the energy demand.

The potential of biomass and solar energy in Pakistan is worked out in many previous studies [9, 10, 11]. However, the literature is deficient in providing evidence on energy security to achieve sustainability. To bridge the existing gap, the objective of the study is twofold. First, the present study analyses the existing energy situation to understand the prevailing energy security and sustainability situation in Pakistan. Second, given the importance of energy security in socio-economic and environment sustainability, the present study empirically examines the short run and long run impact of energy security on socio-economic and environmental sustainability by using Johanson Cointegration and Error Correction model. In meeting these objectives, we provide an evidence to the policy makers to focus on indigenous sources for sustainability and to increase the energy security.

In section two, we focused on situational analysis using descriptive tools. Section three gives methodology to investigate the long run association among energy insecurity, socio-economic condition and environment. Section four covers the estimation and analysis of results. Finally, section five concludes the study and provide policy recommendations.

## 2 Background

### 2.1 Energy supply mix in Pakistan

Pakistan's total primary energy supplies are 70.26 million Tons of Oil Equivalent (TOE). 52.8% of total indigenous production (24.23 million TOE, 34.5% of total demand) is met through net imports (HDIP, 2015). The basic energy products/sources are Natural Gas, Petroleum Products, Liquefied Petroleum Gas, Coal, Hydro, Nuclear and Renewable resources with major reliance on petroleum products and natural gas. Out of total energy supplies, 24.5 million TOE are used in transformation and the remaining 42.7 million TOE are used by domestic, commercial, agriculture, industry, transportation and government sectors.

The share of hydroelectricity and coal in primary energy supplies is only 11% and 7% whereas their shares in electricity generation comprise of 30.4% and 0.1% respectively. Pakistan is also engaged in energy generation based on nuclear (2% of primary energy supplies and 5.4% of electricity generation) and renewable energy resources (0.3% of primary energy supplies and 0.7% of electricity generation). Renewable Energy sources include Solar energy, Biomass, Micro Hydro projects and Wind Energy. However, currently wind power plants are under construction. The percentage share of each source of energy in primary energy supply mix is presented in Table 1. The share of hydrocarbons (Oil and gas) is 78.1%.

**Table 1:** Percentage share of energy sources: Pakistan.

Primary Energy Supply Mix	Percentage Share
Oil	35.5
Gas	42.6
LPG	0.7
LNG	0.7
Coal	7
Hydro-Energy	11
Nuclear-Energy	2
Renewable-Energy (Solar, Biomass & Micro Hydroelectricity)	0.3
Imported-Energy	0.2

**Fig. 1:**
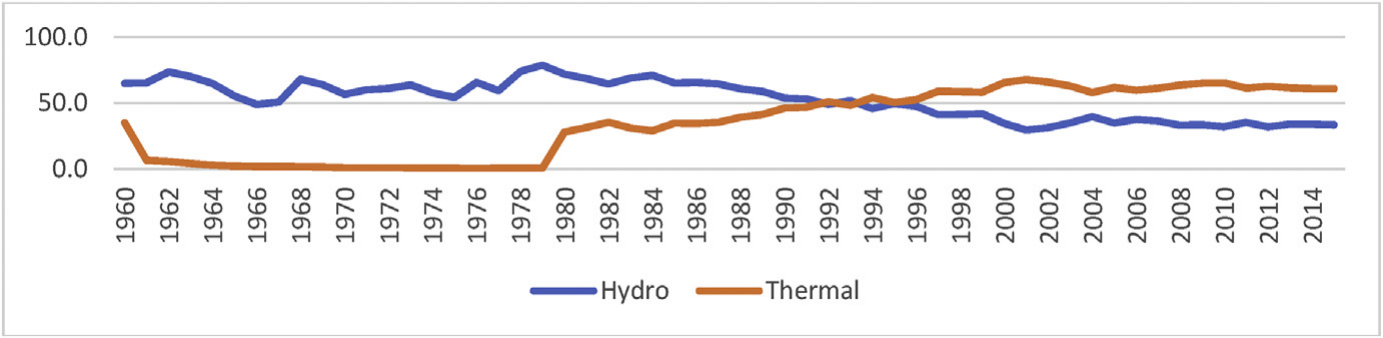
Percentage share of energy generation by hydroelectricity and thermal in Pakistan.

Overtime, Pakistan's reliance on gas and oil resources has increased in comparison to hydroelectricity resources (Fig. 1). The main reason behind this transition is the unexplored potential of hydroelectricity, coal, renewable and nuclear resources for electricity generation. Moreover, during the last three decades, the share of hydroelectricity in primary energy supply has decreased and the share of thermal (oil and gas) resources has increased. However, the share of renewable resources is negligible in the overall energy supplies which calls for attention towards abundant resources of water, coal, solar, wind and biofuels etc.

**Fig. 2:**
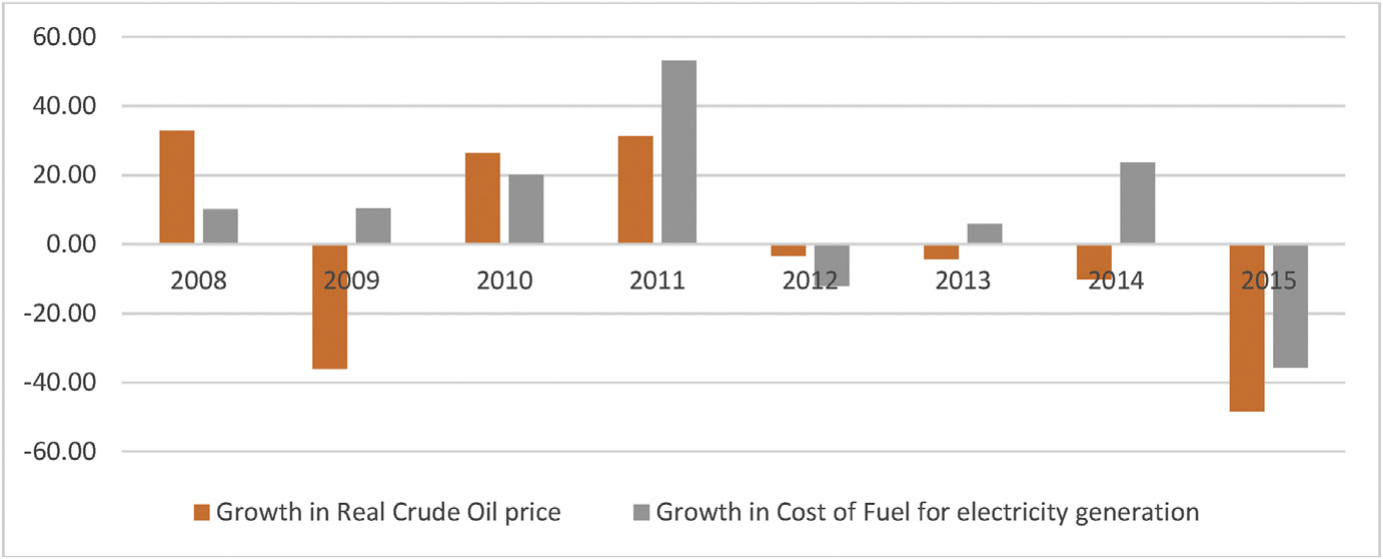
Co-movement of Crude Oil prices and cost of Fuel on electricity.

Sustained supply of affordable and environment friendly energy supply to the population at large is the prime concern of policy makers around the globe. Reliance on expensive resources to produce power is not a rational choice. Oil price variability in the international market put negative pressures on any oil importing economy primarily due to high import cost. The energy sector of Pakistan has been heavily subsidized up till 2008. Afterwards, more deregulated approach and elimination of subsidies by the government of Pakistan resulted in energy price fluctuations in line with the oil price fluctuations in world oil market. Fig. 2 depicts that growth in crude oil prices in the international market results in growth in cost of fuel on electricity generation in Pakistan. Since 2008, growth rate of the cost of fuel for electricity generation has 68% correlation with growth rate of crude oil prices. In this deregulated environment, oil price increases results in rising cost of production. As a result, economy bears high price level, low economic growth and high unemployment, i.e., stagflation. High oil prices have damaging effects on the economy through high inflation and low economic growth accompanied with deteriorating balance of payments and mounting fiscal deficit [12, 13, 14]. High energy prices also erode purchasing power of households because of high energy bills on one hand and low real wages on the other hand [15].

More reliance on resources of energy other than oil can be beneficial both for economic stabilization and competitiveness in the international market. The growing energy needs with increase in population led to increased use of fossil fuels creating many challenges such as greenhouse gas emissions, environmental concerns, depletion of fossil fuels and oil price fluctuations. As also argued by [16], renewable energy sources are the outstanding alternative and the only solution to growing challenges. Increased reliance on fossil fuels cause price fluctuations with the variations in oil prices in the international market which leads to failure of monetary and fiscal authorities to meet the objective of economic stabilization.

In Pakistan, the increased share of thermal power generation is contradictory to world's energy policy. Table 2 explains International energy mix and it depicts that only 4.9% (1.06 kilowatt-hours) of net electricity is generated through liquids in 2012 whereas it is projected to decrease to 1.55% (0.56 kilowatt-hours) by 2040. Reliance on renewables and natural gas is expected to increase up to 29.15% and 27.82% respectively. Though the total quantity of net electricity generation through coal is expected to increase from 8.60 to 10.62 trillion kilowatt-hours but its share will decrease from 39.91% to 29.13%. It calls for attention of the energy policy makers to develop a comprehensive policy for generating electricity through renewables not only for economic sustainability but also for social and environmental sustainability to increase energy security in the country in line with the world energy mix.

**Table 2:** World net electricity generation by energy source, 2010–40 (trillion kilowatt-hours).

	2012	2020	2025	2030	2035	2040
Renewables	4.73	6.87	7.89	8.68	9.64	10.63
Natural gas	4.83	5.26	6.30	7.47	8.78	10.14
Nuclear	2.34	3.05	3.40	3.95	4.25	4.50
Coal	8.60	9.73	10.07	10.12	10.31	10.62
Liquids	1.06	0.86	0.69	0.62	0.59	0.56
Total	21.56	25.77	28.35	30.84	33.58	36.45

### 2.2 Demand and supply gap analysis of electricity

**Fig. 3:**
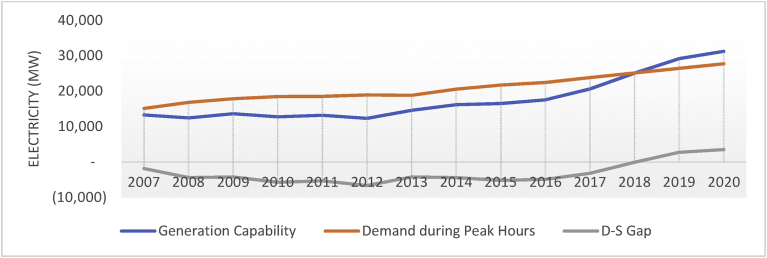
Demand and supply gap of electricity in Pakistan.

Since 2007, another problem in Pakistan is the severe power outages due to demand-supply gap which damaged the social and economic fabric of the country. Generation capability in Pakistan remained stagnant during 2007–2012 which resulted in increasing demand-supply gap from 1.8 GW to 6.6 GW (see Fig. 3 and Table 3). However, from 2013 onwards, generation capability enhanced due to installation of new power projects. The power projects incepted after CPEC have been proven as a major breakthrough to meet the demand-supply gap. After completion of the ongoing power projects, the installed capacity will increase by more than 15,300 MW leading not only to meet the gap but also to meet the country's requirement for the next few years [17]. The current projects however are unlikely to meet the country's requirement in the long run; that is, when CPEC will be in full swing. Further, the electricity projects incepted under CPEC lacks exploring the renewable sources of energy. Accordingly, these do not ensure the economic stabilization, competitiveness, environmental and social sustainability. Thus, there is a need to explore the potential of indigenous energy resources to generate affordable electricity in line with the Pakistan's Vision 2025 and SDGs.

**Table 3:** Capacity, capability and demand of electricity.

Financial Year	Installed Generation Capacity	Generation Capability	Demand during Peak Hours	Demand-Supply Gap (Capability-Peak Demand)	Capacity-Peak Demand
2005	17,367	13,353	12,035	1,318	5,332
2006	17,395	13,402	13,118	284	4,277
2007	17,395	13,292	15,138	(1,846)	2,257
2008	17,526	12,442	16,838	(4,396)	688
2009	17,827	13,637	17,852	(4,215)	(25)
2010	18,022	12,751	18,467	(5,716)	(445)
2011	18,892	13,193	18,521	(5,328)	371
2012	20,986	12,320	18,940	(6,620)	2,046
2013	20,499	14,600	18,827	(4,227)	1,672
2014	20,850	16,170	20,576	(4,406)	274
2015	22,104	16,500	21,701	(5,201)	403

### 2.3 Energy security risk outlook

**Fig. 4:**
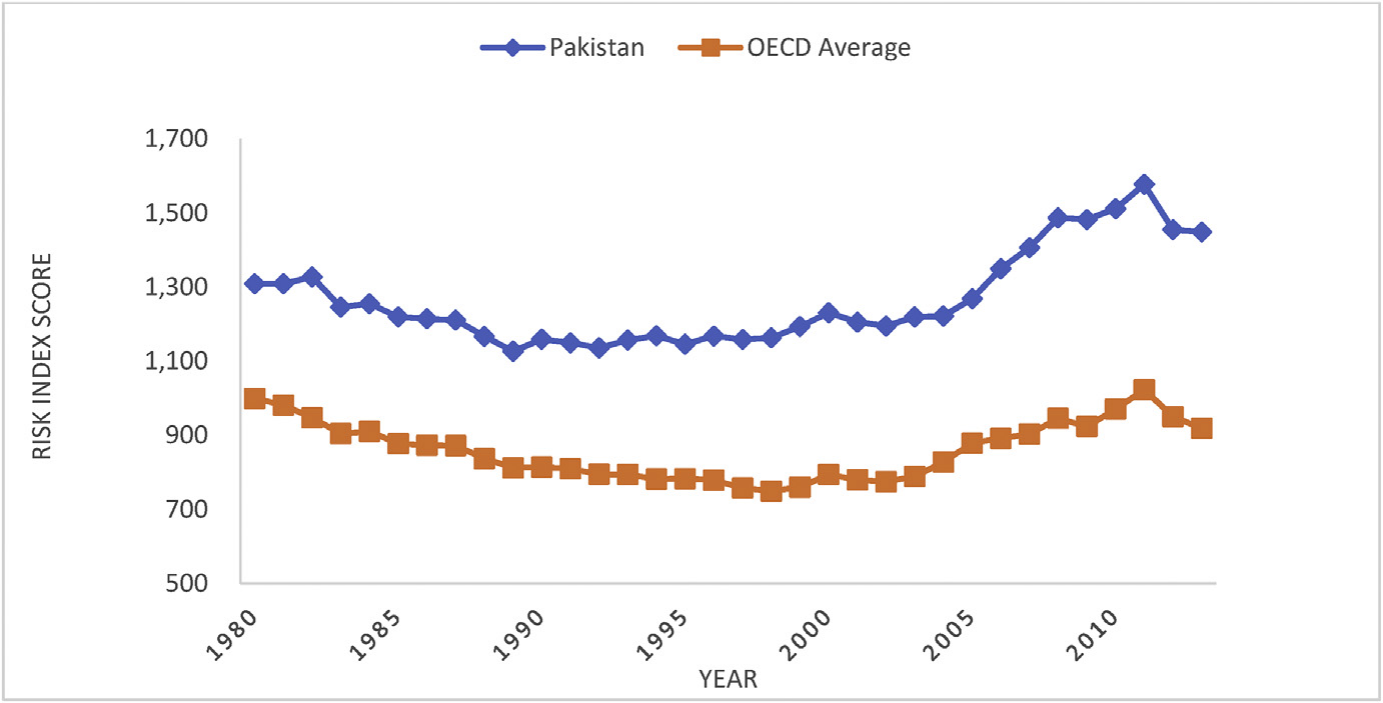
Pakistan vs. OECD: Energy security risk index score.

In Pakistan, exposure to imports of coal, intensity of energy expenditures per GDP, energy intensity, volatility of energy expenditures, energy import expenditures and trend in CO2 emissions are major contributing factors with more than 68% involvement in energy insecurity, according to data on International Index of Energy Security Risk, provided by the Institute of 21st Century Energy, U.S. Chamber of Commerce [18]. The historical pattern of energy security risk index score in Fig. 4 depicts exponential growth in risk wherein Pakistan had ever faced higher risk than OECD countries.

**Fig. 5:**
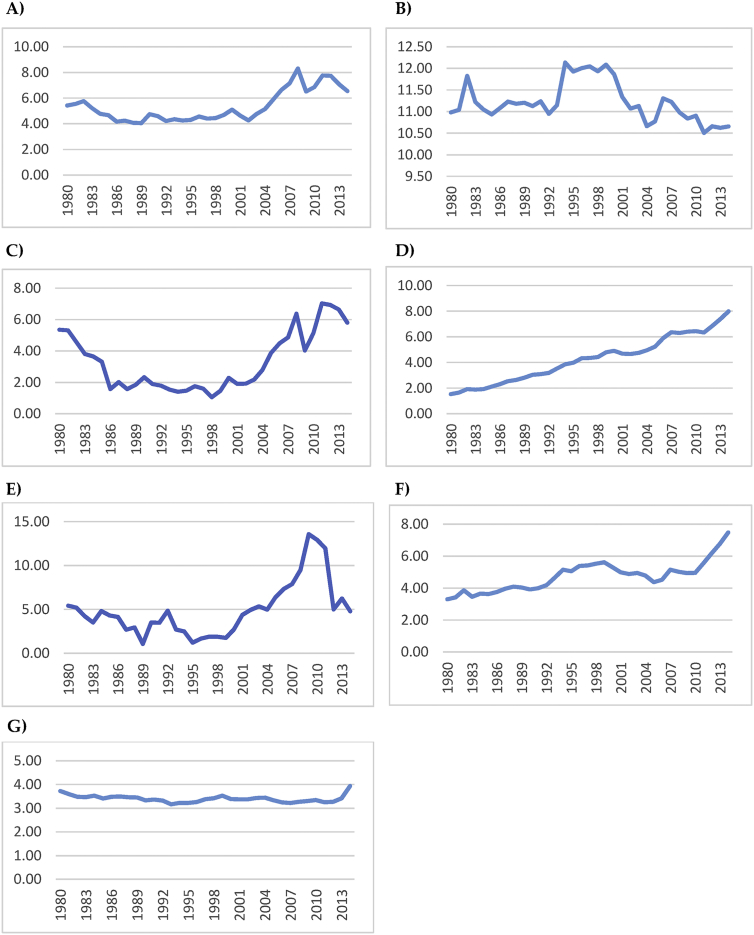
Major factors of energy security risk. A) Energy expenditure intensity, B) Energy intensity, C) Crude oil prices, D) CO2 emissions trend, E) Energy expenditure volatility, F) Transport energy intensity, G) Electricity capacity diversity.

Looking at the various indicators within the index will be more meaningful to understand the major factors behind the rising risk which are depicted in Fig. 5.

### 2.4 Potential of solar and wind sources for electricity generation in Pakistan

Considering the fact that Pakistan has high energy security risk wherein major reliance is on imported hydrocarbons which is damaging for socio-economic and environmental sustainability. Therefore, an important policy direction would be to explore the potential for indigenous renewable sources of energy particularly solar and wind sources in addition to hydroelectricity. Pakistan, especially the southern Punjab, Sindh and Balochistan, have huge solar potential with more than 3 KWH/m^2^/day of radiation for most of days in a year. The available potential is feasible for Concentrated Solar Power (CSP) and Solar Photovoltaic (PV), Off-Grid or On-Grid, with financial and technical viability. The direct solar radiation, having potential of CSP and PV, ranges 5–5.5 KWH/m^2^/day for more than 300 days a year in Southern Punjab. The range in almost all areas of Punjab is 4–6.5 KWH/m^2^/day. Utilizing the available solar potential to produce power not only enable to produce power at lower cost but also to conserve the depleting resources and save foreign exchange. It will also help in combating global warming. Power generation through solar radiations is a viable option having lot of potential which can be explored through installation of small solar power projects at public sector buildings. Industries can also be encouraged to install the solar power projects for self-consumption. Furthermore, there is also need to focus on solar power projects as an option to electrify the villages. Availability of large potential to produce electricity from solar radiations call for on grid and off grid electrification at community level and household level. It can also help in promoting small scale industry in the country.

Ref. [19] presented a case study of a women living in village at Bahawalpur who use solar lamp to sew cloths at night. She became capable enough to meet her household expenditures. Indian Punjab Government, in New and Renewable Sources of Energy Policy [20], put emphasis on Off-grid solar applications, include small powered looms, solar PV pumps, small milk chilling plants, home lighting and hybrid systems for powering telecom towers. Community Development programs, public private partnership or microfinance schemes can be proved as a good source of utilizing solar resources to produce power and fulfil the requirements of households and producers. A solar pumping project was implemented in 2006 for drinking water supplies in six villages of FATA by National University of Science and Technology, with the funding of USAID which can be picked as a good example of solar pumping project. Quaid-e-Azam Solar Power Park, in Cholistan, Bahalpur, has started producing electricity with generating capacity of 400 MW which will be extended to 1000 MW. In addition, various small solar power projects are under construction in many districts like Chakwal, Jehlum, Sialkot, Sahiwal, Bahawalnagar which will bring installation capacity of 242 MW into the national grid. The government of Pakistan can use the solar resources to produce off grid energy to increase access in unelectrified villages and public-sector buildings. The analysis shows that all parts of the country have potential of producing electricity from solar radiations.

Wind energy, the fastest growing renewable source of energy globally, has proven to be the cost competitive and environment friendly source of energy. Steady improvement in technology to produce wind power and performance of wind power plants has been witnessed during the last decade. Developing wind energy primarily depends on wind resources. According to wind map of Pakistan, developed by USAID, Coastal belt of Pakistan has good potential for power generation through wind resources. Pakistan has potential of producing around 346 GW electricity from wind resources available in coastal areas of the country, according to National Renewable Energy Laboratory, United States, currently, installed capacity of Wind power projects is 504.5 MW and number of projects are under construction having capacity of more than 1100 MW, mostly in the Sindh province. Letter of Intent to install a wind power project in southeastern part of the Punjab province, of 50 MW installed capacity, has been issued to AM Energy. In Punjab, more potential is available near Kalar kahar, Chakwal district.

### 2.5 Summary

The above analysis shows that the gap between electricity supply and demand will end before starting year 2019 but it will not change the fuel mix of electricity production. More than 78% of the primary energy supplies depend on hydrocarbons (oil and gas). The increasing reliance on hydrocarbons is not a viable option in socio-economic and environmental perspective as it’s a compromise over sustainability and energy security. Moreover, moving towards efficient use of energy (or through declining energy intensity) can make the country relatively energy secure. Import exposure, energy intensity, electricity diversity, expenditure volatility and CO2 emissions are major trend setters of energy security in the country. Less exposure to coal imports and expenditures on imports lead the country towards security. Both low diversity and high crude oil prices yield high insecurity whereas overall energy intensity, transport energy intensity and energy expenditure intensity results in high insecurity in the country. To increase energy security in the country, Pakistan should focus on less reliance on imported fuel products and need to shift its focus on indigenous energy resources. It is suggested that there is a need to change the energy mix with a shifted focus at provincial level on exploring potential of coal, hydroelectricity, solar and biofuels. It will not only enable to produce power at lower cost but will also enable the producers to become competitive in domestic and foreign markets. The analysis highlights that energy security is required for short run and long run socio-economic and environmental sustainability which is further strengthen through employing rigorous econometric analysis.

## 3 Methodology

### 3.1 Model specification

The energy insecurity (EINS), socio-economic condition (SEC) and environment (ENV) has a direct or indirect relationship with each other for which the functional form is specified in Eqs. (1), (2), and (3).

(1) EINS=f(SEC,ENV)

(2) SEC=f(EINS,ENV)

(3) ENV=f(EINS,ENV)

we are using the regression Eqs. (4), (5), and (6) for the above functional forms:

(4) $$logEINS_{t}=\beta_{0}+\beta_{1}logSEC_{t}+\beta_{2}logENV_{t}+u_{t}$$

(5) $$logSEC_{t}=\beta_{0}+\beta_{1}logEINS_{t}+\beta_{2}logENV_{t}+u_{t}$$

(6) $$logENV_{t}=\beta_{0}+\beta_{1}logSEC_{t}+\beta_{2}logSEC_{t}+u_{t}$$

Where ‘*u*’ is the Gaussian white noise and the subscript “*t”* is a reference for time.

### 3.2 Econometric technique

The literature on time series econometrics suggests that there are chances to get spurious results when the under-consideration data series are non-stationary in nature. Therefore, the researchers may use stationary or differenced variable to overcome this problem. But, the major problem with the differenced data series is that it results in removal of important long run information from the data leaving only the short run information. Therefore, the applied econometricians suggest to determine whether the long run relationship between the variables of interest exists or not and if it exists then the variables can be used at level.

There are a number of techniques available to test the long run association or cointegration between the variables. The most widely used techniques include maximum likelihood based on Johensen [21], the residual based Engel-Granger [22] test, Johensen and Juselius [23] cointegration tests and Auto Regressive Distributed Model estimator. But this study uses the Johensen [21] estimators due to some certain advantages over the other mentioned techniques. For example, one very important issue has to do with the order of the variables. The Engel-Granger [22] test remains silent on which of the variables can be used as regressor. The same problem may occur in the Auto distributed lag model estimators. The second problem is that there is possibility of more than one cointegrating relationships in case there are more than two variables involved. The Engle-Granger procedure using residuals from a single relationship cannot treat this possibility and does not give us the number of cointegrating vector [23]. The third problem is that it relies on a two-step estimator. The first step is to generate the residual series and the second step is to estimate a regression for this series in order to see if the series is stationary or not. Hence, any error introduced in the first step may carried into the second step.

The above mentioned three problems, at least, are resolved in the Johansen's Approach. That's why we are using the Johansen's estimators for this study.

### 3.3 Johansen approach

We have mentioned earlier that if there are more than two variables then there lies possibility of more than one cointegration vector. Therefore, in this case, the Engel-Granger may be inappropriate for estimating the multivariate model [22]. Therefore, an alternative to Engel-Granger is needed which is the Johansen approach for the multiple equations [23].

In order to present this technique, it is convenient to extend the single equation error correction model to a multi-variate model. We have three variables in our estimated model, socio-economic condition (SEC), environment (ENV), and energy insecurity (EINS) and all these three variables can be endogenous. Therefore, we have that in matrix notations as $H_{t}=\mathrm{logSEC},\;\mathrm{EINS},\;\mathrm{ENV}$ and which are represented in Eqs. (7) and (8).

(7) $$H_{t}=B_{1}H_{t-1}+B_{2}H_{t-2}+B_{3}H_{t-3}+\mu$$

(8) $${\text{H}}_{\text{t}}={\text{λ}}_{1}{\text{B}}_{1}{\text{H}}_{\text{t}-1}+{\text{λ}}_{2}{\text{B}}_{2}{\text{H}}_{\text{t}-2}+{\text{λ}}_{3}{\text{B}}_{3}{\text{H}}_{\text{t}-3}+{\text{ΠH}}_{t-1}\text{μ}$$

Where $\lambda_{t}=\left(1-B_{1}-B_{2}-B_{3}\;\right)$ and $\Pi =\;-\left(1-B_{1}-B_{2}-B_{3}\;\right)$.

The ∏ matrix contains the information for the long run.

### 3.4 Vector error correction method (VECM)

When there exists a long run relationship, we are interested in examining whether short run relationship exists or not? These estimates can be obtained by Vector Error Correction Model (VECM) which is restricted Vector Auto Regressive (VAR) and the restriction is the existence of long run relationship. In VECM dependent variable depends on its own lags, the lags of independent variables, the error correction term, and on a random error term. This error term shows how much adjustment is required in the long run.

The basic framework for the analysis is the following model of VECM (comprises of Eqs. (9), (10), and (11)) for three variables.

(9) $$\Delta \text{logEINS}\;=\alpha_{0}+\overset{p}{\underset{i=1}{\sum }}\beta_{1}\Delta logEINS_{t-i\;}\overset{q}{\underset{i=1}{\sum }}\beta_{2}\Delta logSEC_{t-i+}\overset{r}{\underset{i=1}{\sum }}\;\beta_{3}\Delta logENV_{t-i}+\lambda_{1\;}Ec_{t-1}+\mu_{1t}$$

(10) $$\Delta \text{logSEC}\;=\;\gamma_{0}+\overset{p}{\underset{i=1}{\sum }}\beta_{1}\Delta logEINS_{t-i}+\overset{q}{\underset{i=1}{\sum }}\beta_{2}\Delta logSEC_{t-i}+\overset{r}{\underset{i=1}{\sum }}\beta_{3}\Delta logENV_{t-i}+\lambda_{2}Ec_{t-2}+\mu_{2t}$$

(11) $$\Delta \text{logENV}\;=\;\gamma_{0}+\overset{p}{\underset{i=1}{\sum }}\beta_{1}\Delta logEINS_{t-i}+\overset{q}{\underset{i=1}{\sum }}\beta_{2}\Delta logSEC_{t-i}+\overset{r}{\underset{i=1}{\sum }}\beta_{3}\Delta logENV_{t-i}+\lambda_{3}Ec_{t-3}+\mu_{3t}$$

Where $\mu_{\mathrm{it}}$ are serially uncorrelated random error terms. The $Ec_{t-i}$ represents the cointegrating vectors and $\lambda_{i}$ represents the coefficient of adjustment which shows the adjustment of disequilibrium in the previous period. The adjustment coefficient λ has to be statistically significant for the existence of a long-run relationship among the variables.

### 3.5 Granger Causality approach

The correlation between variables does not guarantee in any way that causation also exist. The econometric literature shows that there can be many cases showing correlations in the absence of any theoretical relationship and association, such relationships are considered meaningless or spurious. Granger causality approach is important to test causation and direction of relationship between the variables. We shall also check whether energy insecurity causes socio-economic and environment or reverse causality exists between these three.

### 3.6 Data and variable description

#### 3.6.1 Energy insecurity

Energy is fundamental to the economic development of a society whereas energy security is critical to the socio-economic and environmental sustainability in any country [24]. In the present study, we proxied energy insecurity by energy security risk index. The data is provided by Global Institute of Energy, U.S. Chamber of Commerce for the period 1984–2015. High energy security risk score shows higher energy's volatility and insecurity [18]. Table 4 provides list of indicators included in the energy security risk index along with weighting scheme.

**Table 4:** Indicators of energy security risk index and weighting scheme.

	Metric	Weight
1	Global Oil Reserves	2.0%
2	Global Oil Production	3.0%
3	Global Gas Reserves	2.0%
4	Global Gas Production	3.0%
5	Global Coal Reserves	2.0%
6	Global Coal Production	2.0%
7	Oil Import Exposure	3.0%
8	Gas Import Exposure	3.0%
9	Coal Import Exposure	2.0%
10	Total Energy Import Exposure	4.0%
11	Fossil Fuel Import Expenditure per GDP	5.0%
12	Energy Expenditure Intensity	4.0%
13	Energy Expenditures per Capita	3.0%
14	Retail Electricity Prices	6.0%
15	Crude Oil Prices	7.0%
16	Crude Oil Price Volatility	5.0%
17	Energy Expenditure Volatility	4.0%
18	World Oil Refinery Usage	2.0%
19	GDP per Capita	4.0%
20	Energy Consumption per Capita	4.0%
21	Energy Intensity	7.0%
22	Petroleum Intensity	3.0%
23	Electricity Capacity Diversity	5.0%
24	Non-Carbon Generation	2.0%
25	Transport Energy per Capita	3.0%
26	Transport Energy Intensity	4.0%
27	CO2 Emissions Trend	2.0%
28	CO2 per Capita	2.0%
29	CO2 GDP Intensity	2.0%
	Weighted Total Index	100.0%

#### 3.6.2 Socio-economic condition

The data of socio-economic conditions for the period 1984–2015 is obtained from International Country Risk Guide (ICRG). It is an assessment of the socio-economic pressures at work in society that could constrain government action or fuel social dissatisfaction. The risk rating assigned is the sum of three subcomponents, each with a maximum score of four points and a minimum score of 0 points. A score of 4 points equates to very low risk and a score of 0 points to very high risk. The subcomponents are unemployment, consumer confidence and poverty [25]. Higher the score, the better would be the socio-economic conditions in the country and reciprocally.

#### 3.6.3 Environment

The data on carbon emission from 1984-2015 is obtained from World Development indicator, World Bank. Carbon dioxide (CO_2_) emissions are those stemming from the burning of fossil fuels and the manufacture of cement. They include carbon dioxide produced during consumption of solid, liquid, and gas fuels and gas flaring [26].

## 4 Results and discussion

All the variables are transformed into natural logarithms. The time series properties of the variables or the existence of unit root is checked through employing Augmented Dickey-Fuller (ADF) unit root test [27, 28]. The results of ADF test (see Table 5) suggests the existence of unit root however the unit root of all the variables is removed at first difference, that is, all three variables are integrated of order 1. It reveals the potential of existence of long run relationship among EINS, SEC and ENV.

**Table 5:** Test for unit root.

Variable	ADF t-statistics	P-value
***Levels***		
EINS	−0.882	0.782
SEC	−1.170	0.671
ENV	−0.339	0.907
***1st Difference***		
EINS	−4.815*	0.000
SEC	−6.479*	0.000
ENV	−6.962*	0.000

ADF tests at the 5% and 10% significance levels. * is indicating that null hypothesis is rejected at 5%.

All variables are stationary at 1^st^ difference, meaning thereby, we can apply the Johansen cointegration test to analyze the long run association between these variables. Johansen's cointegration test is determining that of the number of cointegration vectors at the first r where we fail to reject the null hypothesis.

The results of the Johansen cointegration tests based on ECM using an optimal lag length of two are provided in Table 6. The results from both maximum Eigen-value tests and the trace indicate that cointegration exists among the variables in the system. In this situation when long run relationship (cointegrating relationships) exists among variables, ECM specification is appropriate. Furthermore, the residuals of the ECM specification are white noise [29].

**Table 6:** Johansen cointegration test results.

Cointegrating rank			
***Cointegration Rank Test (Trace)***			
	Eigenvalue	Trace Statistics	Probability
r = 0	0.565∗	49.594∗	0.034
r ≤ 1	0.294	21.244	0.342
r ≤ 2	0.156	9.399	0.329
r ≤ 3	0.101	3.623	0.057
***Cointegration Rank Test (Max. Eigenvalue)***			
	Eigenvalue	Max. Eigen Statistics	Probability
r = 0	0.565∗	28.349∗	0.039
r ≤ 1	0.294	11.845	0.563
r ≤ 2	0.156	5.776	0.642
r ≤ 3	0.101	3.623	0.057

r denotes the number of cointegrating vectors.

∗ Denotes rejection of the null hypothesis of cointegration rank r at the 5% significance level. It is indicating that log-run association exist between these variables.

Table 7 presents the results of ECM based Granger causality tests. Equation of each of the variables is presented in columns. For each of the variables, there exists one channel of causality at least: either the short-run Granger causality which can be verified through joint significance tests (F-test) of the lagged-differenced coefficients or the long-run causality that can be confirmed through statistically significant lagged error-correction term (t-statistics). The coefficient of lag of ECT is significant which implies that past equilibrium errors affect current outcomes. In this case, energy insecurity Granger cause socio-economic condition and environment in the short run but socio-economic and environment does not Granger cause energy insecurity in the short run. Both the hypotheses are supported by the data at 10% level of significance. The error terms are statistically significant. The first one indicates that social economic conditions and energy insecurity jointly cause environment damages in the long run. Second error term is indicating that environment and energy insecurity jointly cause socio-economic condition in the long run. These conditions do not hold reversely in the long run.

**Table 7:** Short run and long run F-test results based on error correction models (ECM).

Short run Granger Causality F-test results			
Variables	EINS	SEC	ENV
EINS	-	1.436 (0.258)	5.358 (0.012)
SEC	1.159 (0.331)	-	3.203 (0.033)
ENV	2.949** (0.072)	4.565** (0.057)	-

**Long run Granger causality test results**			

ECT (Dependent EINS)	−0.799* (0.005)	0.724 (0.044)	−0.542 (0.158)
ECT (Dependent SEC)	−0.445* (0.002)	−1.13* (0.016)	0.402 (0.039)
ECT (Dependent ENV)	−0.991 (0.201)	1.020 (1.110)	−0.161 (0.451)

P-values are in parentheses. * and ** denotes at 5% and 10% significance level.

## 5 Conclusion

Global agenda to meet the energy security is primary concern of the world today. During the last four decades, the world has changed its focus from imported expensive energy resources to cheaper renewable sources that also bring socio-economic and environmental sustainability. Diversification of energy resources along with optimal utilization of indigenous resources must be a part of national strategy. Instead of focusing on the indigenously available energy resources, Pakistan has increased reliance on imported hydrocarbons for production of electricity which does not has environmental consequences only but also results in lowering competitiveness and increasing social hazard. Even during the last few years, heavy investments on coal and LNG power projects have been witnessed to mitigate the electricity shortage in the country. With the on-going policy of increasing reliance on imported energy sources, it would be hard to achieve sustainable Development Goal 7 and Pakistan's Vision 2025, that is, to ensure reliable, affordable and accessible energy. Despite abundant solar and wind resources, the unwanted electricity shortfall has affected Pakistan's economy since 2007. The increase in demand for energy is natural due to increase in economic activity and population which call for optimizing the energy mix through increased reliance on hydroelectricity, indigenous coal, solar, wind and biomass. It will ensure lesser volatility and lower electricity price.

Ensuring energy security is also important for short run and long run socio-economic and environmental sustainability. There exists long run association among energy security risk, environment and socio-economic conditions. Socio-economic conditions and energy security risk jointly cause environmental damages in the long run. Environment and Energy security risk also jointly cause deterioration in socio-economic conditions. Through employing Granger Causality F-test based on Error Correction Model, short terms causality of energy security risk to environment and socio-economic conditions is also evident.

The results suggest that Pakistan should focus on indigenously available resources which are rich like Solar, Wind in coastal areas and water in Khyber Pakhtunkhwa and Azad Jammu & Kashmir so that energy security risk can be minimized and environmental and socio-economic conditions can be improved. This policy will ensure better environmental and socio-economic conditions for the future generations and fulfilment of Sustainable Development Goal 7 would not be a difficult task then. Moreover, the analysis shows that the on-going demand-supply gap of electricity would end before the start of 2019 but it will not change the fuel mix to produce electricity. Therefore, there is need to develop a long run energy policy which may ensure energy security in the country. Considering the availability of solar radiations for production of electricity, it is suggested that government may provide off-grid and on-grid solutions to the community to fulfil their demand of electricity. Microfinance models for installation of solar panels at household level can help to mitigate the growing demand through off-grid electricity production.

## Declarations

### Author contribution statement

Shahzada M. Naeem Nawaz, Shahzad Alvi: Conceived and designed the experiments; Performed the experiments; Analyzed and interpreted the data; Contributed reagents, materials, analysis tools or data; Wrote the paper.

### Funding statement

This research did not receive any specific grant from funding agencies in the public, commercial, or not-for-profit sectors.

### Competing interest statement

The authors declare no conflict of interest.

### Additional information

No additional information is available for this paper.
